# Subjective clinical visual disturbance scores correlation with simulated vehicle headlight halo in presbyopia-correcting intraocular lenses

**DOI:** 10.1007/s00417-026-07197-1

**Published:** 2026-04-17

**Authors:** Thomas Kohnen, Jessie M. Hull, Asrar Alkrizy, Vidhyapriya Sreenivasan, Daniel Carson

**Affiliations:** 1 Department of Ophthalmology, Goethe-University Frankfurt, Theodor-Stern-Kai 7, Frankfurt am Main, 60590 Germany; 2 Alcon Vision LLC, Fort Worth, TX USA

**Keywords:** Diffractive EDOF IOLs, Nondiffractive EDOF IOLs, Halos, Monofocal IOLs, Multifocal IOLs, Presbyopia-correcting IOLs

## Abstract

**Purpose:**

To evaluate the correlation between objective bench assessment of halos and subjective clinical experience in patients receiving monofocal and presbyopia-correcting intraocular lenses (IOLs).

**Methods:**

Data from four clinical studies included patients with cataracts who received bilateral monofocal (SN60AT, SN60WF), extended depth-of-focus (EDOF; ZXR00, DFT015, 829MP), or multifocal (ZFR00V, TFNT00) IOLs. Subjective halo severity was assessed 6 months after implantation using the Questionnaire for Visual Disturbance (QUVID). Bench assessments were measured using a simulation of a 100-mm headlight at 31 m (20,000 cd/m^2^ intensity) and an IOL mounted in a model eye with a 4.5-mm pupil. The area under the curve (AUC) of intensity plot was calculated for each IOL and compared with the QUVID-based assessment using a statistical regression analysis. Correlation was assessed using linear regression analysis.

**Results:**

In clinical studies, moderate to severe halos were reported by 29% to 48% of patients with diffractive (TFNT00, ZXR00, 829MP, ZFR00V), 11% with nondiffractive EDOF (DFT015), and 4% to 7% with monofocal (SN60WF and SN60AT) IOLs. Diffractive IOLs produced larger halos (AUC, 1448–1896) compared with nondiffractive EDOF (997) and monofocal (1002–1008) IOLs. Correlations were reported between bench halo AUC and patient-reported severity (no halo, *r* = − 0.92; little to mild halo, *r* = 0.43; moderate to severe halo, *r* = 0.93).

**Conclusions:**

A greater proportion of patients with diffractive versus nondiffractive IOLs reported severe halos. Bench halos correlated with subjective clinical experience, particularly for moderate and severe halos.

## Abstract (highlights)

**Key messages:**

***What is known*:**

- Advances in IOL design, such as wavefront-shaping nondiffractive technology, aim to provide functional spectacle-independent near, intermediate, and distance vision while minimizing the risk of visual disturbances associated with multifocal IOLs.

***What is new*:**

- Correlations were reported between bench halos assessed using a simulated vehicle headlight and patient-reported halo severity, particularly for patients with moderate to severe halos.
- Bench assessments of halos could be used to predict subjective halo severity experienced by patients.

## Introduction

Presbyopia-correcting intraocular lenses (IOLs) can be used to replace the natural lens and help achieve spectacle independence in patients after cataract and refractive lens exchange surgery [1, 2]. Although monofocal IOLs can provide excellent distance vision, spectacles are typically required for near and intermediate distances [1, 2]. Multiple types of presbyopia-correcting IOLs designed to provide functional uncorrected vision over a range of distances are currently available, including multifocal and extended depth-of-focus (EDOF) IOLs [3].

Multifocal IOLs can have diffractive or refractive technology that enables patients to focus on images at multiple focal planes, aiming to provide simultaneous vision at multiple distances and reduce the need for spectacles [4, 5]. However, multifocal IOLs are associated with patient-reported visual disturbances, such as halos, glare, and starbursts [2, 6]. Multifocal IOLs, such as the AcrySof IQ PanOptix IOL (TFNT00; Alcon Research LLC, Fort Worth, TX, USA), have multiple addition powers (i.e., at 40 and 60 cm) to help achieve near, intermediate, and far vision [7]. Both clinical and bench studies reported that multifocal IOLs, including AcrySof IQ ReSTOR (SN6AD1; Alcon Research LLC), Tecnis ZKB00 (Johnson and Johnson, New Brunswick, NJ, USA), Tecnis Synergy ZFR00V (Johnson and Johnson), and PanOptix IOLs, were associated with halos [8, 9].

Extended depth-of-focus IOLs were developed to provide an extended range of vision from distance to intermediate, while maintaining a better quality of distance vision compared with multifocal IOLs [10–13]. Wavefront-shaping nondiffractive technology can be used to provide the desired range of vision without the increased risk for visual disturbances [10]. The wavefront-shaping IOL AcrySof IQ Vivity (DFT015; Alcon Research LLC) was shown to improve distance and intermediate vision and reduce spectacle dependence compared with monofocal IOLs, with rates of visual disturbances similar to those with monofocal IOLs [14, 15]. In bench studies, wavefront shaping and refractive EDOF IOLs demonstrated halos similar to those with monofocal IOLs [9, 16, 17].

Some EDOF IOLs rely on nondiffractive optics to extend the range of vision, whereas others achieve EDOF with a multifocal design [18]. The Tecnis Symfony ZXR00 IOL (Johnson and Johnson) uses diffractive step-like optics that extend the range of vision [19]. The AT LARA EDOF IOL (829MP; Zeiss Meditec AG, Jena, Germany) has a diffractive anterior surface with low addition powers to enable continuous visual acuity from far to intermediate-near distances [20]. Similar to multifocal IOLs, these diffractive EDOF IOLs have been associated with halos in clinical and bench studies [9, 21].

The Tecnis Synergy ZFR00V IOL uses hybrid technology that combines diffractive multifocal and diffractive EDOF to provide intermediate and near vision and high patient satisfaction, although it has also been associated with halos [9, 22–24].

Predicting visual disturbance is desirable when designing a new presbyopia-correcting IOL and selecting IOLs for patients. Evaluating halos using simulated headlights and bench assessments can be a useful tool during the new lens design process. However, interpretation of halos measured in bench studies is limited by the laboratory setup and may not be directly applicable to clinical practice. Understanding the correlation between bench studies and patient-reported visual disturbances is important for providing a robust approach to quantitative assessment of halos and comparison of halos among different IOL designs.

The purpose of this study was to evaluate the correlation between an objective bench assessment of halos and the subjective clinical experience of patients from standardized questionnaires for monofocal and presbyopia-correcting IOLs, including diffractive and nondiffractive wavefront-shaping optical designs.

## Methods

### Subjective clinical experience

This analysis used data from 4 clinical studies in patients who underwent cataract surgery and received one of the following IOLs bilaterally (Table 1): AcrySof spherical monofocal IOL (SN60AT; Alcon Research LLC; *n* = 114), AcrySof IQ aspheric monofocal IOL (SN60WF; Alcon Research LLC; *n* = 109), AcrySof IQ PanOptix trifocal IOL (TFNT00; *n* = 267), AcrySof IQ Vivity extended vision IOL (DFT015; *n* = 168), Tecnis Symfony diffractive EDOF IOL (ZXR00; *n* = 60), AT LARA diffractive EDOF IOL (829MP; *n* = 61), and Tecnis Synergy diffractive EDOF IOL (ZFR00V; *n* = 138) [14, 25, 26]. Enrolled patients were adults with bilateral cataracts and planned routine cataract surgery. Preoperative CDVA was 0.3 logMAR (20/40 Snellen) or worse and preoperative astigmatism was < 1.0 D in both eyes. Calculated lens power was within 15.0–26.5 D, 18.0–25.0 D, or 15.0–25.0 D, depending on the specific study. All studies excluded patients with disease or pathology expected to reduce potential postoperative CDVA to be worse than 0.2 logMAR.

**Table 1:** Intraocular lenses included in the analysis

IOL Model	Manufacturer	Category	Optical Principle
AcrySof Spherical SN60AT	Alcon	Monofocal	Nondiffractive
AcrySof Aspheric SN60WF	Alcon	Monofocal	Nondiffractive
AcrySof IQ PanOptix TFNT00	Alcon	Trifocal	Diffractive
AcrySof IQ Vivity DFT015	Alcon	EDOF	Nondiffractive wavefront shaping
Tecnis Symfony ZXR00	Johnson and Johnson	EDOF	Diffractive
AT LARA 829MP	Carl Zeiss Meditec	EDOF	Diffractive
Tecnis Synergy ZFR00V	Johnson and Johnson	EDOF/ multifocal	Diffractive

*EDOF* Extended depth of focus, *IOL* Intraocular lens

**Fig. 1:**
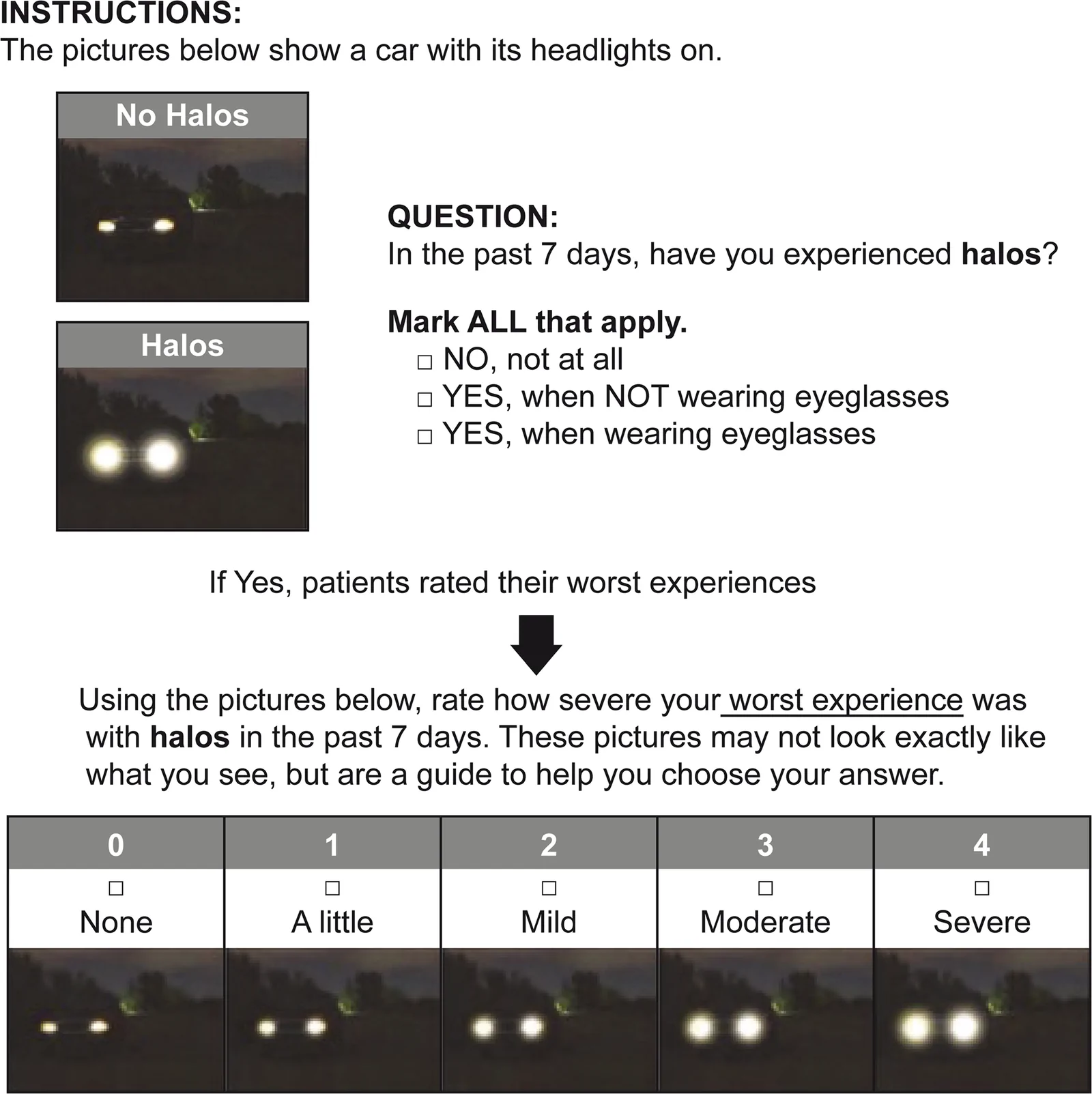
The Questionnaire for Visual Disturbance developed and validated to collect patient-reported outcomes on visual disturbances

Subjective clinical halo experience was assessed postoperatively at 6 months in patients with visual acuity better than 0.2 logMAR using the validated Questionnaire for Visual Disturbance [27] (QUVID; Fig. 1). QUVID was developed to assess visual symptoms, including halos, in patients with cataracts. The questionnaire assessed frequency, severity, and bothersomeness of 7 visual symptoms in the past 7 days measured using a 5-point Likert-type scale [27]. Halo severity was subjectively rated by patients as *none*, *a little*, *mild*, *moderate*, or *severe*.

### Bench assessments

**Fig. 2:**
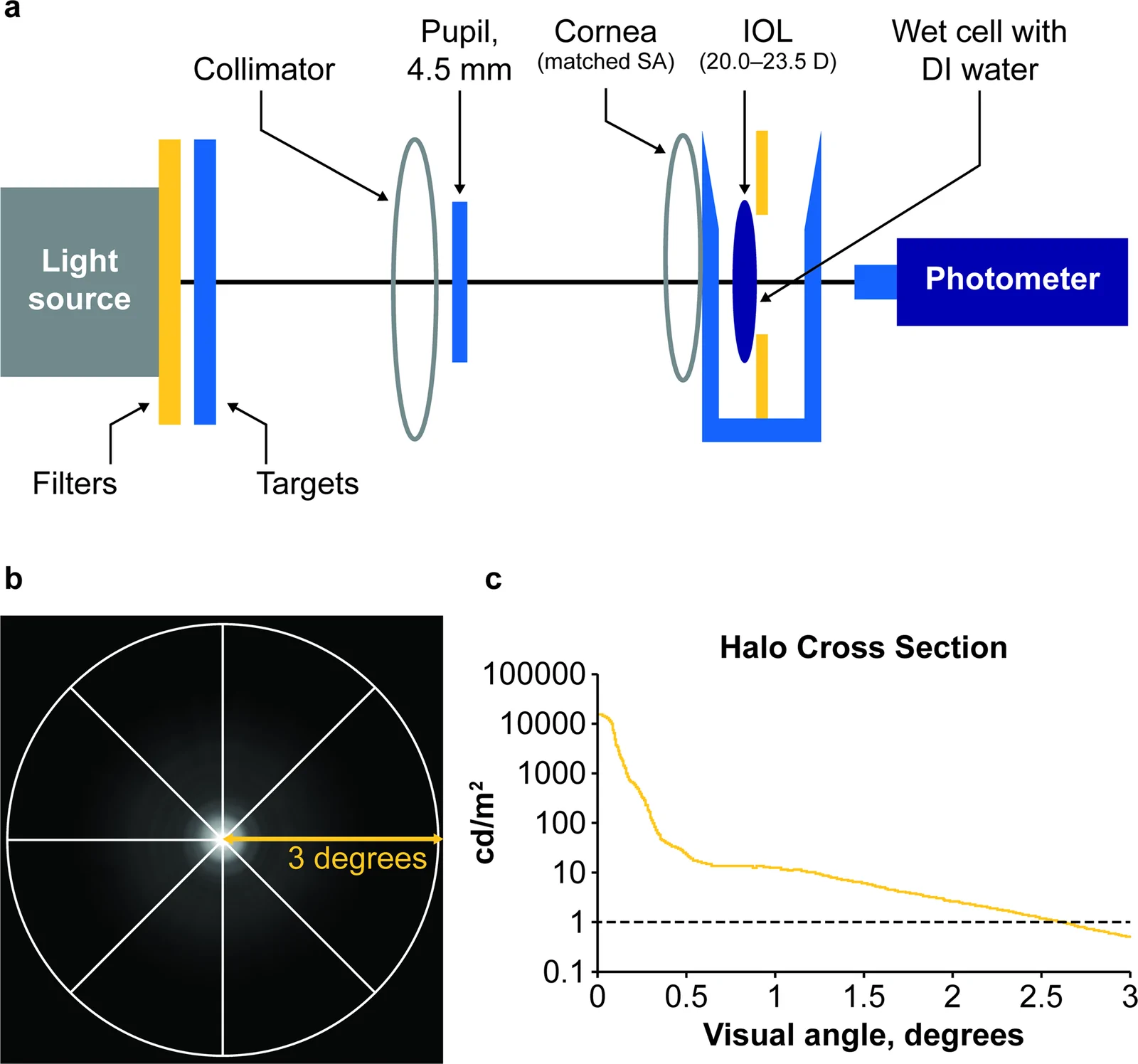
Bench assessments diagrams. Bench setup simulating a vehicle headlight to assess the halo produced by various IOLs (**a**). Bench halos were measured using the high dynamic range system with a pinhole simulating a 100-mm headlight at 31 m. Eight radial profiles of a bench halo image (**b**) were averaged over 6° field of view, and the AUC was calculated from the area under the profile curve (**c**). The sample shown is IOL model ZFR00V. AUC, area under the curve; D, diopter; DI, deionized; IOL, intraocular lens; SA, spherical aberration. The diagrams were used without changes from previously published materials by Kohnen et al. 2023 [9] licensed under Creative Commons Attribution 4.0 International License (https://creativecommons.org/licenses/by/4.0/)

Objective assessment was conducted using a bench set-up simulating a vehicle headlight to assess the halo produced by various IOLs (Fig. 2A). Bench halos were measured using the high dynamic range camera system. A pinhole was illuminated with a broad-spectrum light-emitting diode (LED) light source to simulate a 100-mm diameter headlight at 31 m. IOLs were mounted in a model eye with matched corneal spherical aberration and an external pupil scaled to 4.5 mm at the IOL plane. A 4× microscope objective relayed the image from the model eye to the camera. Light intensity was set just below saturation with a monofocal IOL, about 20,000 cd/m^2^. Using the camera image of the pinhole, a plot was made of the luminance intensity as a function of visual angle. For each IOL, the area under the curve (AUC) of the intensity plot was computed from the image center to a visual angle of 3°. The ALOHA software (Alcon Research LLC) computed the AUC of every logarithmic image, calculated from the average intensity cross-section to an extent of 3°, starting at the center of the pinhole image along 8 lines 45° apart (Fig. 2B and C). The AUC calculation is given in arbitrary units because the vertical axis is normalized, with the peak luminance values at 0 normalized to 1.

### Correlation of bench and subjective assessments

For all IOL models, the AUC halo profile calculated from bench assessments was compared with the subjective QUVID-based halo severity assessment using a statistical regression analysis. For each IOL, a single AUC value was plotted against 3 levels of halo severity presented as *none*, *little/mild*, and *moderate/severe*. Linear regression analysis was used to assess the correlation between AUC and halo severity. Both unweighted correlation analysis and weighted correlation analysis by sample size (per IOL model) were used.

## Results

**Fig. 3:**
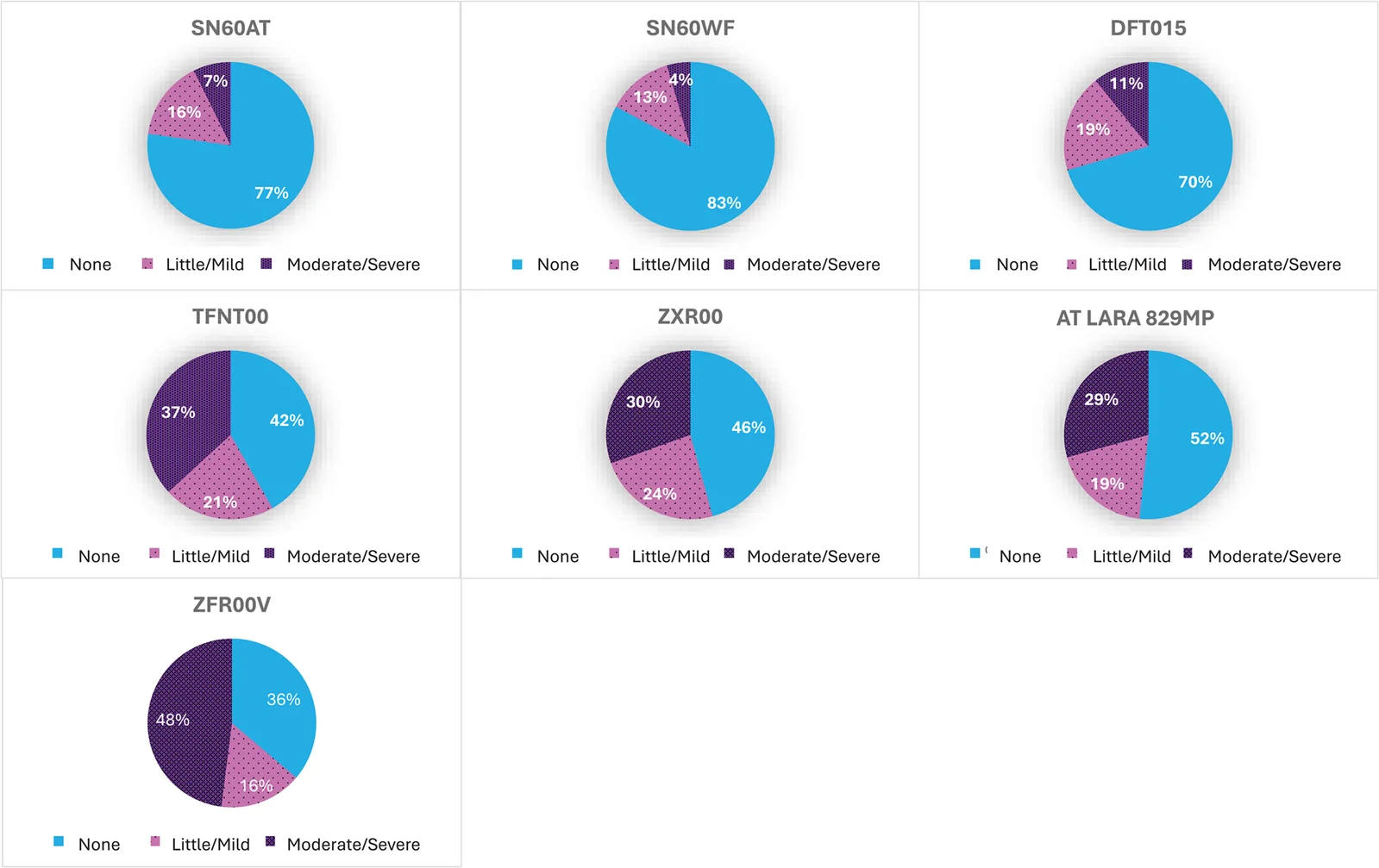
Clinical experience of halos using QUVID data from patients. IOL, intraocular lens; QUVID, Questionnaire for Visual Disturbance

Data from QUVID showed that moderate to severe halos were reported by 29% to 48% of patients who received diffractive IOLs (TFNT00, ZXR00, 829MP, ZFR00V), 11% of patients with wavefront-shaping nondiffractive IOLs (DFT015), and 4% to 7% of patients with monofocal IOLs (SN60WF and SN60AT; Fig. 3). A lower percentage of patients with nondiffractive IOLs (SN60WF, SN60AT, DFT015) reported moderate to severe halos when rating their worst halo experience compared with patients who received diffractive IOLs (4% to 11%; Fig. 3).

**Fig. 4:**
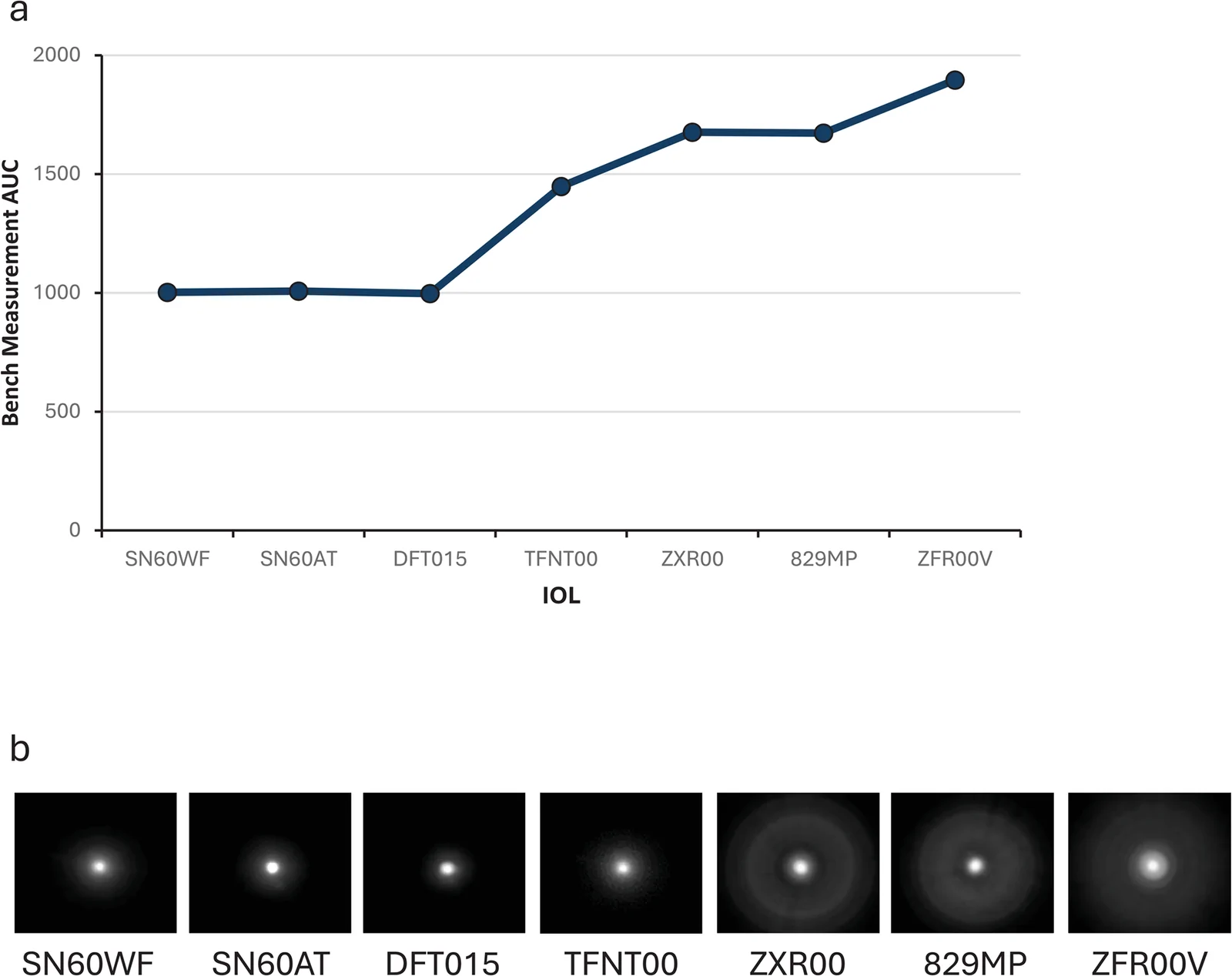
Bench halo AUC measurements for monofocal, wavefront-shaping, and diffractive IOLs (**a**) and bench halo images (**b**). AUC, area under the curve; IOL, intraocular lens

Bench halo AUC demonstrated that diffractive IOLs (TFNT00, ZXR00, 829MP, ZFR00V) produced larger halos compared with monofocal and nondiffractive wavefront-shaping IOLs (SN60WF, SN60AT, DFT015). AUC values were 1448 to 1896 for diffractive, 997 for nondiffractive, and 1002 to 1008 for monofocal IOLs (Fig. 4A). Bench halo images are shown in Fig. 4B.

**Fig. 5:**
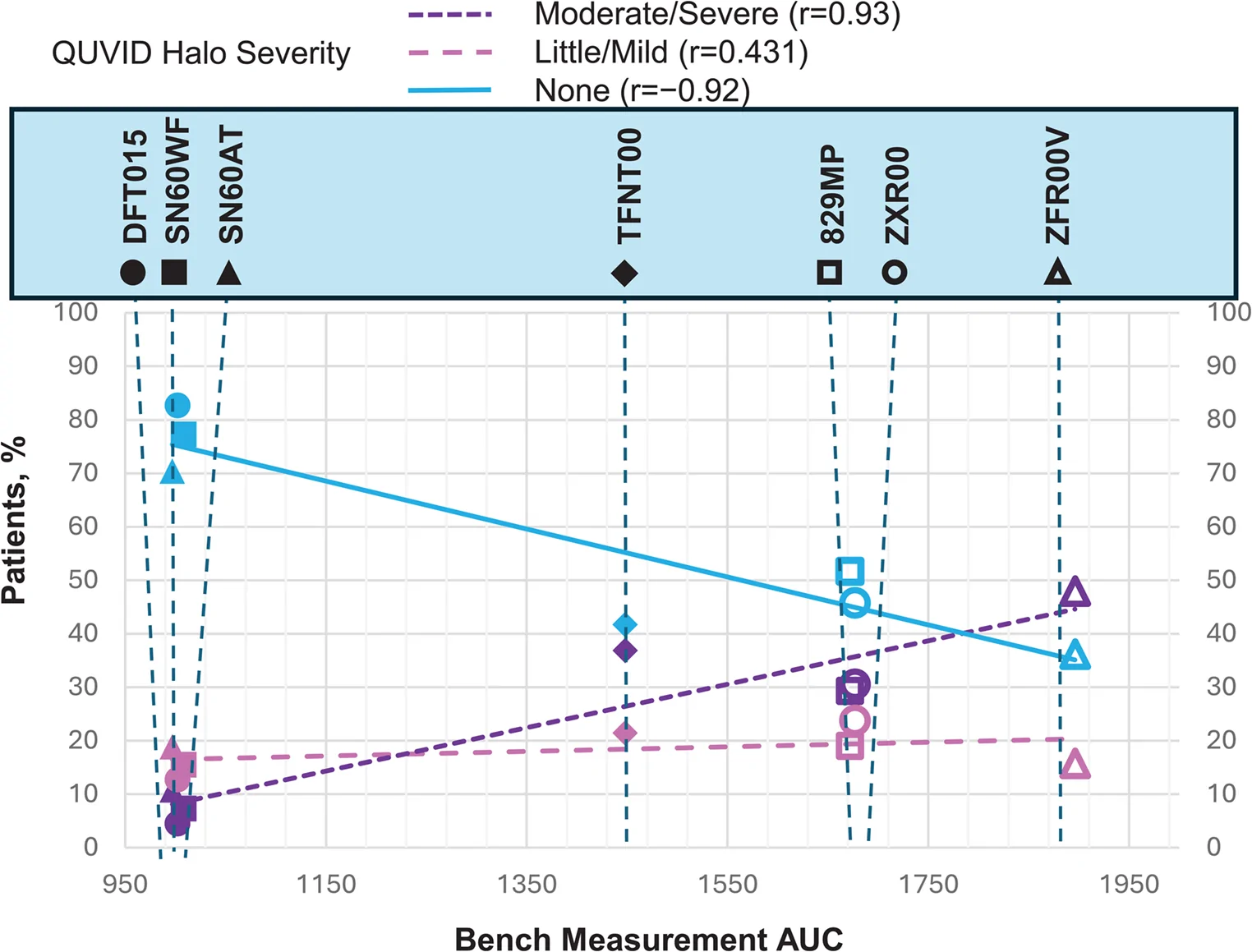
Correlation between bench halo AUC and patient-reported QUVID halo severity. For each IOL type, laboratory-measured AUC was plotted against the halo severity reported clinically. Halo severity is presented as none (blue), little/mild (pink), and moderate/severe (purple). AUC, area under the curve; IOL, intraocular lens; QUVID, Questionnaire for Visual Disturbance

Correlations between bench halo AUC and patient-reported halo severity were moderate to high (Fig. 5). Based on linear regression, the correlation was higher for moderate to severe halo (unweighted *r* = 0.93; weighted *r* = 0.94) or no halo (unweighted *r* = − 0.92; weighted *r* = − 0.92) than for little to mild halo (unweighted *r* = 0.43; weighted *r* = 0.38; Fig. 5), with AUC of bench halo being positively correlated with rates of patient-reported moderate to severe halo and negatively correlated with rates of patient’s reporting no severe halo.

## Discussion

This study evaluated clinical reports and bench assessments of halos for monofocal and presbyopia-correcting IOLs. The results of the validated QUVID data analysis demonstrated that a greater proportion of patients with diffractive IOLs reported moderate to severe halos compared with nondiffractive wavefront-shaping and monofocal IOLs. Furthermore, there was a high correlation (*r* = 0.93) between bench measurements assessing halos and rates of patient-reported moderate to severe halos.

Presbyopia-correcting IOLs, such as multifocal and EDOF IOLs, have been developed to provide more spectacle-independent vision and to improve patient satisfaction. However, multifocal IOLs have been associated with a higher risk of visual disturbances, such as halos [2]. Visual disturbances occurring after IOL implantation can affect patient quality of life after cataract surgery [28]. Most notably, halos can negatively affect nighttime driving [29, 30]. Other presbyopia-correcting IOLs, such as those with EDOF based on generating a single elongated focal point, were designed to enhance intermediate and near vision while minimizing visual disturbances [18]. Results of the current study provide additional evidence of reduction in halos for nondiffractive wavefront-shaping EDOF IOLs (DFT015) compared with diffractive presbyopia-correcting IOLs (TFNT00, ZXR00, AT LARA 829MP, and ZFR00V).

These findings are consistent with previous bench studies that used objective laboratory methods to assess and quantify halos. In a bench study that simulated nighttime light sources and modeled the human eye during nighttime driving conditions, the smallest halos were observed with monofocal IOLs (AcrySof IQ SN60WF) compared with multifocal IOLs; the greatest halos were observed with Tecnis Multifocal (ZKB00) and Tecnis Symfony EDOF IOL (ZXR00) [31]. There was a strong correlation between bench assessments and subjective questionnaire-based halo assessments for mild (*r* = 0.98) and severe (*r* = 0.93) halos based on linear regression [31]. Another bench study similarly reported that monofocal (SN60WF) and wavefront-shaping (DFT015) IOLs had minimal light spread, whereas diffractive IOLs (ZXR00 and 829MP) had an extended halo pattern [17].

In a separate bench study that evaluated optical performance, ZXR00 had similar halo intensity when compared with multifocal IOLs Tecnis ZMB00 and FineVision GFree (PhysIOL, Liege, Belgium) [19]. Similarly, a bench study that evaluated and compared halos produced by monofocal and presbyopia-correcting IOLs reported that diffractive IOLs had larger halos compared with monofocal IOLs and nondiffractive wavefront-shaping IOLs [9].

Among the strengths of this study was the use of QUVID for assessing patient-reported visual disturbances. The QUVID was developed and validated based on guidance from the US Food and Drug Administration to reliably capture patient-reported outcomes for visual symptoms. The QUVID was based on literature and instrument review, multiple clinician and patient interviews, assessment of psychometric properties, and assessment of the capability to detect meaningful change [27]. The correlation between QUVID and bench assessments of halos suggest that bench measurements could be used to predict clinical subjective responses on severity of visual disturbances. The observed negative correlation between bench halo AUC and the proportion of patients reporting no halo was consistent with the expected clinical interpretation of the AUC metric. A greater bench halo AUC reflected a more pronounced halo profile, which corresponded to a greater proportion of patients reporting halo symptoms and, consequently, a lower proportion of patients reporting no halo. This pattern was also based on the categorical nature of the patient-reported outcomes, where the QUVID categories of “none,” “little/mild,” and “moderate/severe” would add up to 100% within each IOL group. Weighted and unweighted analyses were consistent and suggest that the observed associations were robust and not dependent on sample size imbalances across IOL models. The interpretation is limited by the small number of IOLs studied with QUVID providing data points to support this correlation. Furthermore, bench halos may not explain other common symptoms, such as starbursts or glare. An additional limitation of this analysis was that data for correlating pupil size and mesopic contrast sensitivity were not available for all the studies.

Although bench measurements are not a direct evaluation of the halo severity that patients may experience, these assessments provide useful information that can describe and clarify optical performance and visual disturbances associated with IOLs. Specifically, bench assessments provide a reliable way to measure halo severity and allow a quantitative comparison of optical performances of currently available and novel IOLs. Because some patients with cataracts may prefer full range of vision (i.e., multifocal IOLs), even if they experience visual disturbances, future studies assessing the correlation among range of vision, visual disturbances, and patient-reported satisfaction are of great interest. Future studies could also address the correlation between halo severity and functional visual outcomes such as uncorrected distance, intermediate, and near visual acuity.

In conclusion, subjective halo assessments demonstrated that severe halos were experienced by a greater proportion of patients with diffractive IOLs versus nondiffractive IOLs. Bench halo assessments were highly correlated with clinical experience, especially for patients reporting moderate or severe halos. These results suggest that bench studies could be a useful method of predicting clinical responses on severity of halos.

## Data Availability

Data are available from Alcon Vision LLC and can be provided upon reasonable request.
